# Effects of a Short-Term Resistance-Training Program on Heart Rate Variability in Children With Cystic Fibrosis—A Randomized Controlled Trial

**DOI:** 10.3389/fphys.2021.652029

**Published:** 2021-03-30

**Authors:** Agustín Jesús Estévez-González, Márcio Vinícius Fagundes Donadio, Fernando Cobo-Vicente, Álvaro Fernández-Luna, Verónica Sanz-Santiago, José Ramón Villa Asensi, Tamara Iturriaga Ramirez, Maria Fernández-del-Valle, Ignacio Diez-Vega, Eneko Larumbe-Zabala, Margarita Pérez-Ruiz

**Affiliations:** ^1^Faculty of Sport Sciences, Universidad Europea de Madrid, Madrid, Spain; ^2^Laboratory of Pediatric Physical Activity, Centro Infant, Pontifícia Universidade Católica do Rio Grande do Sul (PUCRS), Porto Alegre, Brazil; ^3^Pulmonology Unit, Hospital Universitario Niño Jesús de Madrid, Madrid, Spain; ^4^Department of Applied Health, Southern Illinois University (SIUE), Edwardsville, IL, United States

**Keywords:** heart rate variability, exercise, resistance, autonomic function, pediatrics, cystic fibrosis

## Abstract

**Background:** Cystic fibrosis (CF) affects the autonomic nervous system (ANS) and exercise in healthy children modulates the interaction between sympathetic and parasympathetic activity. This study aimed to evaluate the effects of a short-term resistance exercise program on heart rate variability (HRV) in children and adolescents with CF.

**Methods:** A randomized controlled trial was carried out in children diagnosed with CF aged 6–18 years. Individuals were divided into two groups: control (CON) and resistance-training (EX). Individuals in the EX group completed an individualized guided resistance program (5-RM—60–80%) for 8 weeks (3 sessions of 60 min/week). Upper and lower limbs exercises (seated bench press, seated lateral row, and leg press) were used. HRV was measured using a Suunto watch with subjects in lying position.

**Results:** Nineteen subjects (13 boys) were included (CON = 11; and EX = 8). Mean age was 12.2 ± 3.3, FEV_1_ (forced expiratory volume in the first second) z-score was 1.72 ± 1.54 and peak oxygen consumption (VO_2_peak) 42.7 ± 7.4 mL.Kg^–1^.min^–1^. Exercise induced significant changes in the frequency-domain variables, including a decrease in LF power (*p* = 0.001, *d* = 0.98) and LF/HF ratio (*p* = 0.020, *d* = 0.92), and an increase in HF power (*p* = 0.001, *d* = −0.97), compared to the CON group. No significant changes were found for time-domain variables, although increases with a moderate effect size were seen for SDNN (*p* = 0.152, *d* = −0.41) and RMSSD (*p* = 0.059, *d* = −0.49) compared to the CON group.

**Conclusion:** A short-term resistance exercise-training program was able to modulate HRV in children and adolescents with CF presenting mild to moderate lung function impairment and good physical condition.

**Clinical Trial Registration:** www.ClinicalTrials.gov, identifier NCT04293926.

## Introduction

Cystic fibrosis (CF) is an autosomal recessive disease caused by the mutation of the cystic fibrosis transmembrane conductance regulator (CFTR) gene, located in the chromosome 7 (Wainwright et al., 1985). The defect of the chlorine channel affects all epithelial cells, altering ionic transport in several tissues and causing obstruction of the secretory glands (Savant and McColley, 2019). Although CF is a multisystemic disease, pulmonary impairment is the most frequent symptom, as a result of small airway obstruction that triggers a chronic inflammatory process (Savant and McColley, 2019). However, the effects of the CFTR malfunction on the autonomic nervous system (ANS), as well as its physiological consequences, still remain to be better understood.

CFTR dysfunction may lead to abnormalities in the ANS (Davis and Kaliner, 1983), as it facilitates neuronal activity, as well as plays an important role in the regulation of energy homeostasis, motor function and autonomic control of visceral organs, such as the heart (Kahle et al., 2008). Several studies suggest that ANS could be altered in CF (Davis and Kaliner, 1983). A simple and non-invasive method to analyze the function of ANS is to record the sympathetic-parasympathetic balance measured through heart rate variability (HRV). This method allows a non-invasive overview of the nervous system. HRV is highly sensitive as its negative variations may anticipate autonomic neuropathy clinical symptoms, gaining importance as an useful tool for diagnosis (Malik et al., 1996). In recent years, HRV complexes, as time-domain and frequency-domain values, have generated interest on health science studies and applications based on ANS changes have been used on training programs (Grant et al., 2011). Evidence shows an excellent reproducibility of HRV in healthy children, indicating that this may be an useful tool to evaluate the effectiveness of interventions in this population, as well as a prognostic tool in children with CF (McNarry and Mackintosh, 2016). Early dysfunction of the ANS, measured through HRV, has been described for children with CF (Florêncio et al., 2013; McNarry and Mackintosh, 2016). In addition, in adults with CF, a significant association between HRV, lung impairment and clinical symptoms, such as pulmonary exacerbations, gastrointestinal motility or cough have been reported (Reznikov, 2017).

Exercise in healthy children can positively modulate the ANS (Winsley, 2002). Although there are no studies assessing the effect of exercise on HRV in individuals with CF, there is evidence that exercise may positively influence HRV in obese (Prado et al., 2010) and asthmatic (McNarry et al., 2019) children. Children with CF are characterized by exercise intolerance and higher perceived fatigue, resulting in higher sedentary habits that may negatively impact the evolution and prognosis of the disease. In individuals with CF, the CFTR protein is not expressed in the muscles (Divangahi et al., 2009), leading to cellular alterations that include the oxidative metabolism (Antigny et al., 2009), and reduction of contractile excitability (Clay, 2013), translating into exercise intolerance and decreased muscle strength (Gruet et al., 2017), which contributes to a decreased level of activities, causing a worst response of the ANS. Although there is evidence on the use of aerobic exercise in CF (Radtke et al., 2017), the relative benefits of resistance training for children with CF has been poorly investigated to date (Kriemler et al., 2013; Santana-Sosa et al., 2014).

Thus, the aim of the present study is to determine the effects of a short-term resistance-training program on HRV in children and adolescents with CF. We hypothesize that a resistance exercise-training program would improve the sympatho-vagal balance in individuals with CF.

## Materials and Methods

### Study Design

**FIGURE 1:**
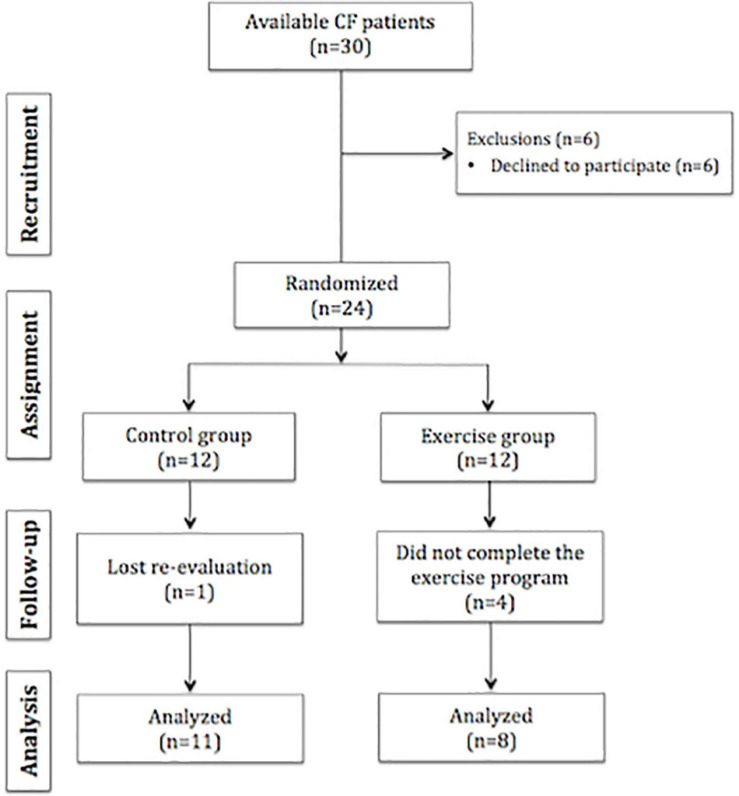
Flow chart of the study.

A randomized controlled trial was conducted in a sample of children and adolescents diagnosed with CF presenting mild to moderate pulmonary impairment. After the screening, participants were randomly assigned to one of the following groups: Control Group (CON) or Exercise Group (EX). All participants undertook two assessments performed by the same investigators: (i) before the intervention (PRE); and (ii) after (POST) the intervention (8 weeks of training) was completed (Figure 1). The study adhered to CONSORT guidelines (Moher et al., 2012) and all experimental procedures were conducted in accordance with the Declaration of Helsinki. Before the start of the study, the protocol was approved by both the University Research Ethics Committee (number CIPI/18/050) and the Hospital Research Ethical Committee (number CI R-001918).

### Subjects

The study started with a convenience sample of all individuals with CF (*n* = 30) presenting a positive genetic test, mild to moderate pulmonary impairment, who met the study inclusion criteria, and were regularly followed at a specialized CF center. The inclusion criteria were described as: (i) children and adolescent aged 6–17 years; (ii) mild to moderate lung function impairment; and (iii) absence of pulmonary exacerbations in the previous 6 months. The exclusion criteria included: (i) presence of percutaneous endoscopic gastronomy; (ii) being under use of drugs that may alter the ANS function (β-blockers, atropine and others); (iii) lung transplant; (iv) active smoking; (v) presence of any skeletal muscle problem that would interfere with either the intervention program or the physical evaluation; (vi) presence of any other pulmonary or cardiac disease that would cause exercise-induced symptoms.

### Randomization

Participants were randomly assigned to one of the experimental groups (CON or EX). The researcher who analyzed the data and the physician responsible for the clinical evaluation were blinded to group assignment of the subjects. As the intervention was based on an exercise program, it was impossible to blind the researchers involved on the training protocol, as well as the participants. Subjects and their parents or legal guardians were informed about the assignment to each group.

### Clinical Anamnesis

Genetic, demographic data and clinical information were collected, at baseline, from medical records or directly with subjects, including: age, sex, comorbidities (hepatic disease), pancreatic sufficiency and bacteriological colonization of the airways.

### Anthropometric Evaluation

Weight and height were measured using a mechanic scale (Asimed, Barys Plus C) and a stadiometer (Seca 213, OMRON), respectively. Body mass index (BMI) was calculated by the ratio between weight and squared height (Kg/m^2^). Raw values were adjusted for age and sex using z-scores according to the WHO reference values (World Health Organization, 2018).

### Lung Function

Lung function was assessed through spirometry, using a Master Screen (Jaeger, Germany), following American Thoracic Society—European Respiratory Society (ATS/ERS) guidelines. Forced vital capacity (FVC), forced expiratory volume in the first second (FEV_1_), FEV_1_/FVC ratio and forced expiratory flow between 25 and 75% of FVC (FEF_25__–__75__%_) were evaluated. Data were expressed as absolute values and z-score, normalized using the Global Lung Initiative (GLI) reference equation (Quanjer et al., 2012). Lower limit of normal (LLN) was set at −1.64 for the FEV_1_ z-score values.

### Cardiopulmonary Exercise Testing

Cardiorespiratory fitness was assessed using a treadmill (Technogym Run Race 1400HC; Gambettola, Italy) maximum test. The incremental protocol used began with an initial speed and inclination of 2.5 km/h and 0.5%, respectively, with increases of 0.1 km/h and 0.5% every 15 s (Santana Sosa et al., 2012). Gas exchange data were measured breath-by-breath through open-circuit spirometry (Vmax 29C; SensorMedics; Yorba Linda, CA) using specific pediatric facemasks, electrocardiogram (ECG) recording and peripheral oxygen (SpO_2_) monitoring (GE Datex Ohmeda TruSat). Peak oxygen consumption (VO_2_peak), peak heart rate (HRpeak), minute ventilation at peak (V_E_peak) and the respiratory exchange ratio (RER) were measured. The VO_2_peak was recorded as the highest value obtained for any continuous 20 s period (Santana Sosa et al., 2012). The test was considered as maximum if the following criteria were achieved: (i) HR greater than 180 beats per minute; and (ii) RER above 1.0.

### Muscle Strength Measurement

The 5 repetition maximum test (5RM) for leg press, seated bench press and seated bilateral row was performed in pediatric machines (Strive Inc., PA, United States) to evaluate peripheral muscle strength. The 5RM values are defined as the maximum strength capacity to perform five repetitions until momentary muscular exhaustion and were measured in kilograms (kg). Before the start of the testing protocol, one session was used to explain the correct technique of the exercises. The testing protocol consisted of three warm-up sets, separated by 1 min rest period, at 50, 70, and 90% of the perceived 5RM. After a 2- to 3-min resting period, 5RM attempt was made at 100% of the perceived effort (Santana Sosa et al., 2012). The final values obtained were normalized by dividing each data by the body weight of the subject. In order to adequately prescribe the training dose, values obtained from 5RM test were used to calculate 1RM using the Bryzicki equation (Abdul-Hameed et al., 2012). Training load doses used in the exercise program were individualized and based on the percentage of the estimated 1RM.

### HRV Recordings

HRV measurements were obtained using a HR band and an Ambit 3 Sport watch (Suunto^®^, Vantaa, Finland). A short-term HRV measurement protocol was used to register 5 min HRV data in supine position without movement or speaking (Abhishekh et al., 2013). Electrical signals were recorded at room temperature (24°C) and with controlled relative humidity (30%). For data analysis, only stable R-R intervals were used and an automated low artifact correction was applied (Kubios software), with adjustment of up to 5%, and ectopic beats eliminated in manual filtration. Baseline respiratory rate was registered. Time-domain variables measured were: standard deviation of R-R intervals (SDNN) in milliseconds (ms); root mean square of successive differences between normal heartbeats (RMSSD) in ms; and percentage of successive R-R intervals that differ by more than 50 ms (PNN50). Frequency-domain variables evaluated were: low frequency (LF) band (0.04–0.15 Hz) in normal units (nu); high frequency (HF) band (0.15–0.40 Hz) in nu; and the ratio between both frequencies (LF/HF ratio). All HRV variables were chosen following international standard guidelines of measurement and its clinical relevance to investigate the ANS (Malik et al., 1996). Data were computed using Kubios HRV analysis software (Biosignal Analysis and Medical Imagine Group, University of Easter Finland, Finland).

### Exercise Intervention Program

Before the start of the study, all participants underwent a familiarization period with all the tests for outcome assessment. The familiarization period included one session for treadmill testing and two 30-min sessions for functional/strength tests. All familiarization, testing, and training sessions took place in the afternoon/evening (4:00–7:00 p.m.). The resistance-exercise training was conducted for 8 weeks, three exercise sessions per week (60 min each) with a minimum interval between sessions of 24 h, divided in: warm-up (15 min), resistance exercises (35 min) and cool-down (10 min). Resistance exercises included both upper limb (bilateral seated row and seated bench press) and lower limb (leg press, leg extension and leg flexion) (Supplementary Figure 1). All exercises were performed in specific pediatric machines. Each session was performed individually, under strict hygienic control (as recommended for individuals with CF) and supervised by an exercise-training professional with a 1:1 supervisor-to-subject ratio. The resistance protocol was divided into two phases: (i) initial phase—performed at 60–70% of 1RM, as previously described, using 8–12 repetitions and 3 series, focused on technique and execution (length of 2 weeks); and (ii) final phase—performed at 70–80% of 1RM (6–8 repetions × 3 series) focused on strength (length of 6 weeks). Control group followed the habitual recommendations of the CF multidisciplinary team. All patients received the same orientations during the routine clinic visit in order to maintain disease control, including routine nutritional and physical activity advisement.

### Statistical Analysis

Data distribution and normality were assessed using Shapiro-Wilk test and diagnostic plots. Categorical data were summarized as frequency (percentage) and continuous data were summarized as mean (standard deviation). Differences between groups in categorical outcomes were assessed using chi-squared (χ^2^) test or Fisher’s exact test, as appropriate. For each continuous outcome, raw difference (D) from PRE to POST with 95% confidence interval (95% CI) was calculated, and statistical significance was assessed using Student’s *t*-test for one sample. In order to compare the effect of the intervention with control from PRE to POST, percent change was calculated as (POST − PRE)/PRE × 100. Differences between groups in percent change were assessed using Student’s *t*-test for independent samples. A two-way analysis of variance (ANOVA) with repeated measures was used to compare absolute differences between groups (CON and EX) and times (PRE and POST) for all HRV outcomes. Significance level was set at 0.05. Standardized effect sizes were reported using Cohen’s d. The interpretation of effect sizes was done according to standard references (Cohen, 1988). All statistical analyses were performed with Stata 15.1 (StataCorp, College Station, TX).

## Results

A total of 30 individuals were assessed for eligibility, between March and September 2020, and 6 refused to participate. Therefore, 24 were randomized (CON = 12 and EX = 12), but one subject in the control group and four in the exercise group were lost to follow-up. Figure 1 shows the flow diagram of the study.

The final sample presented differences regarding genotyping (*p* = 0.032), as the percent of F508del heterozygous was higher in the CON group. Apart from that, no statistically significant differences between groups were found at baseline regarding demographic, clinical, lung function and microbiological variables. As for anthropometric measurements, although significant differences were found for absolute values of body weight and BMI, when variables were normalized in z-scores, no differences were found (Table 1).

**TABLE 1:** Characteristics of the study sample.

	**Control**	**Exercise**	**Total**	***p***
	**(*n* = 11)**	**(*n* = 8)**	**(*n* = 19)**	
***Demographics***				
Sex, n (%)				>0.999
Male	8 (72.7)	5 (62.5)	13 (68.4)	
Female	3 (27.3)	3 (37.5)	6 (31.6)	
Age (years), mean (SD)	11.7 (3.5)	12.8 (3.1)	12.2 (3.3)	0.517
Range	7–19	8–17	7–19	
***Anthropometrics*, mean (SD)**				
Weight (kg)	34.4 (7.9)	44.1 (10.3)	38.5 (10.0)	**0.034**
Weight (z-score)	−1.04 (1.23)	−0.24 (1.18)	−0.7 (1.25)	0.171
Height (cm)	143.5 (13.5)	152.3 (15.1)	147.2 (14.5)	0.199
Height (z-score)	−0.77 (1.15)	−0.27 (0.89)	−0.56 (1.05)	0.321
BMI (kg/m^2^)	16.4 (1.6)	18.8 (2.6)	17.4 (2.4)	**0.021**
BMI (z-score)	−0.83 (1.10)	−0.10 (1.34)	−0.52 (1.23)	0.207
***Genotyping*, n (%)**				
F508del homozigous	1 (9.1)	4 (50.0)	5 (26.3)	**0.032**
F508del heterozygous	9 (81.8)	2 (25.0)	11 (57.9)	
Other mutations	1 (9.1)	2 (25.0)	3 (15.8)	
***Lung Function*, mean (SD)**				
FEV_1_ (L)	1.7 (0.3)	2.2 (0.7)	1.9 (0.6)	**0.038**
FEV_1_ (z-score)	−1.98 (1.49)	−1.37 (1.63)	−1.72 (1.54)	0.405
FVC (L)	2.1 (0.4)	2.9 (1.1)	2.4 (0.9)	0.060
FVC (z-score)	−1.47 (1.43)	−0.75 (1.68)	−1.17 (1.54)	0.327
FEV_1_/FVC	79.7 (10.7)	79.4 (9.5)	79.6 (10.0)	0.956
FEV_1_/FVC (z-score)	−1.05 (1.32)	−1.09 (1.23)	−1.07 (1.25)	0.937
***Cardiorespiratory fitness*, mean (SD)**				
HRpeak (bpm)	182.9 (12.5)	185.0 (7.7)	183.8 (10.5)	0.681
VO_2_peak (mL.Kg^–1^.min^–1^)	43.5 (7.3)	41.7 (8.1)	42.7 (7.4)	0.615
V_E_peak (L.min^–1^)	52.9 (16.2)	66.2 (26.7)	58.5 (21.6)	0.198
***Clinical diagnoses*, n (%)**				
Pancreatic insufficiency	10 (90.9)	7 (87.5)	17 (89.5)	>0.999
Hepatic disease	2 (18.2)	1 (12.5)	3 (15.8)	>0.999
***Microbiologic data*, n (%)**				
Allergic bronchopulmonary aspergillosis	2 (18.2)	0 (0)	2 (10.5)	0.485
Pseudomonas aeruginosa	8 (72.7)	6 (75.0)	14 (73.7)	0.890
Staphylococcus aureus sensible	10 (90.9)	6 (75.0)	16 (84.2)	0.240
Staphylococcus aureus resistant	2 (18.2)	3 (37.5)	5 (26.3)	0.762
Burkholderia cepacia	0 (0)	3 (37.5)	3 (15.8)	0.058
Mycobacterium abscessus	1 (9.1)	0 (0)	1 (5.3)	>0.999

*FEV_1_, forced expiratory volume in the first second; FVC, forced vital capacity; VO_2_, oxygen consumption; HR, heart rate; V_*E*_, minute ventilation; L, liters; Kg, kilograms; SD, standard deviation. Differences between groups were calculated using Student’s t-test, chi^2^-test or exact test, as appropriate. Bold values indicate significant differences (*p* < 0.05).*

The resistance exercise-training program induced a significant increase on muscle strength (bench press, pectoral, and dorsal) when compared to the CON group, demonstrating the effectiveness of the program (Table 2). As for cardiorespiratory fitness (CPET), a significant difference between groups (*p* = 0.019) was found only for the HR at 2 min of recovery. Although VO_2_ at the anaerobic threshold (AT) and VCO_2_ at AT presented moderate effect-size differences in percent change between groups (*d* = −0.49 and *d* = −0.58, respectively), these differences were not statistically significant (Table 2).

**TABLE 2:** Between-group comparisons in percent change from pre to post for cardiorespiratory fitness and muscle strength variables.

	**Control**	**Exercise**	**Total**	***p***	***d***
	**(*n* = 11)**	**(*n* = 8)**	**(*n* = 19)**		
***Cardiorespiratory fitness*, mean (SD)**					
VO_2_peak (mL.Kg^–1^.min^–1^)	−0.2 (11.1)	−2.8 (11.7)	−1.3 (11.1)	0.626	0.24
V_E_peak (L.min^–1^)	−0.6 (11.2)	−5.5 (20.0)	−2.7 (15.2)	0.505	0.32
VO_2_ at AT (%)	−11.2 (15.7)	−4.8 (9.4)	−8.4 (13.3)	0.321	−0.49
VCO_2_ at AT	−19.3 (18.8)	−8.8 (15.9)	−14.6 (17.9)	0.228	−0.58
HR2min recovery (bpm)	2.0 (6.2)	−8.5 (10.6)	−2.7 (9.8)	**0.019**	1.07
***Muscle strength*, mean (SD)**					
Bench press	17.8 (36.7)	167.7 (82.3)	80.9 (95.7)	**0.000**	−1.57
Pectoral	4.6 (16.9)	28.1 (17.4)	14.5 (20.5)	**0.009**	−1.15
Dorsal	12.6 (10.9)	52.1 (28.8)	29.2 (28.1)	**0.001**	−1.40

*VO_2_, oxygen consumption; V_*E*_, minute ventilation; VCO_2_, carbon dioxide production; AT, anaerobic threshold; HR, heart rate; Kg, kilograms; L, liters; bpm, beats per minute; SD, standard deviation. Differences were assessed using Student’s t-test for independent samples. Bold values indicate significant differences (*p* < 0.05). Standardized effect sizes were reported using Cohen’s d.*

**FIGURE 2:**
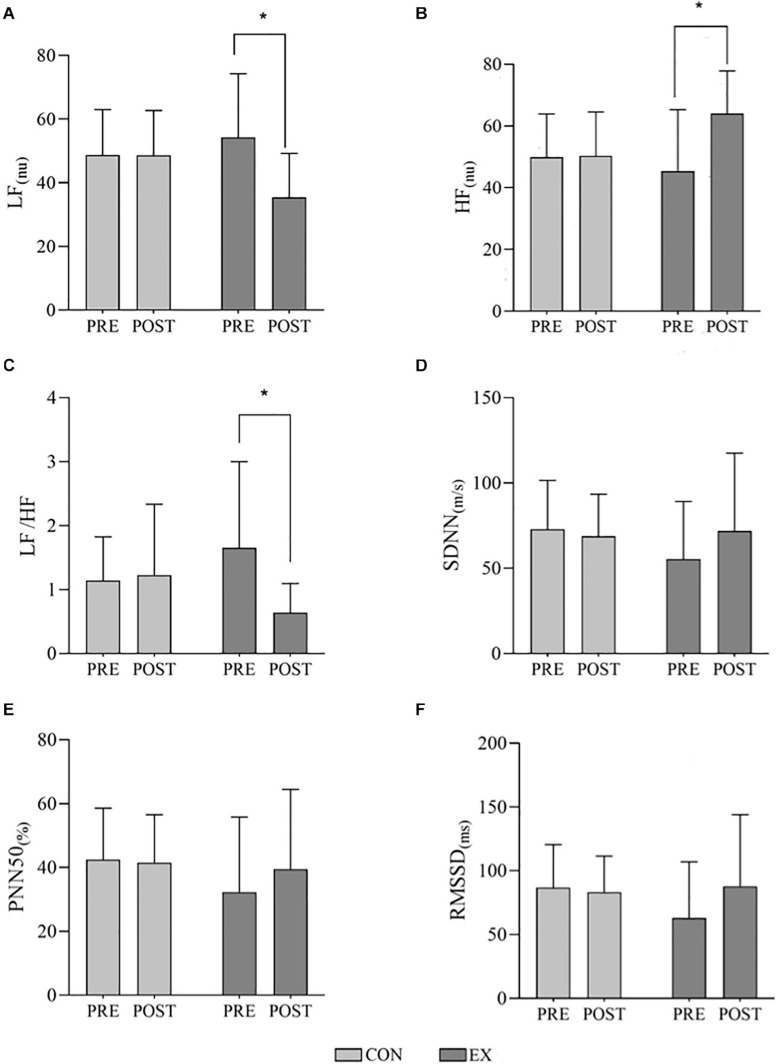
Effects of a short-term resistance-training program on heart rate variability (HRV) in children with cystic fibrosis. **(A)** LF, low frequency band; **(B)** HF, high frequency band; **(C)** LF/HF ratio; **(D)** SDNN, standard deviation of R-R intervals; **(E)** PNN50, percentage of successive R-R intervals that differ by more than 50 ms; and **(F)** RMSSD, root mean square of successive differences between normal heartbeats. ms, milliseconds; nu, normalized units; CON, control group; EX, exercise group. Data presented as mean and standard deviation. Differences were analyzed using a two-way analysis of variance (ANOVA) with repeated measures. *Indicates significant differences when *p* < 0.05.

When HRV data were normalized, according to reference values (Gasior et al., 2018), 57.9% of subjects presented at least one HRV variable classified as altered. No differences in baseline respiratory rate was found between groups (Pre: CON = 25.2 ± 7.4; EX = 27.5 ± 8.6, and Post: CON = 23.6 ± 5.0; EX = 25.5 ± 7.8; *p* = 0.801). Figure 2 presents mean and standard deviation for main HRV data. While no statistically significant pre-to-post differences in HRV were determined for the control group, the exercise group presented large effect sizes and statistically significant differences in frequency-domain metrics (LF power, HF power and LF/HF ratio). Moderate and small effect sizes were also observed in time-domain and non-linear measurements for the exercise group (Table 3). Accordingly, differences between control and exercise groups in percent change were found to be statistically significant in LF power (*p* = 0.006, *d* = 1.20), HF power (*p* = 0.025, *d* = −1.01) and LF/HF ratio (*p* = 0.007, *d* = 1.17). Although SDNN and RMSSD also presented large effect-size differences in percent change between groups (*d* = −0.81 and *d* = −0.84, respectively), these differences were not statistically significant.

**TABLE 3:** Heart rate variability within-group raw differences and between-group comparison in percent change.

	**Control (*n* = 11)**			**Exercise (*n* = 8)**			**Between groups**	
			
	**D [95% CI]**	***p***	***d***	**D [95% CI]**	***p***	***d***	***p***	***d***
SDNN (ms)	−4.15 [−19.80; 11.49]	>0.999	0.16	16.44 [−1.91; 34.78]	0.152	−0.41	0.080	−0.81
RMSSD (ms)	−3.38 [−22.16; 15.40]	>0.999	0.11	24.84 [2.81; 46.86]	0.059	−0.49	0.068	−0.84
PNN50 (beats)	−0.94 [−10.82; 8.94]	>0.999	0.06	7.18 [−4.41; 18.76]	0.417	−0.30	0.282	−0.51
LF (ms^2^)	383.6 [−710.3; 1477.4]	0.453	0.78	475.1 [−111.9; 1062.2]	0.097	1.91	0.806	0.12
LF (nu)	−0.11 [−8.24; 8.03]	>0.999	0.01	−18.79 [−28.33; −9.25]	**0.001**	0.98	**0.006**	1.20
HF (ms^2^)	−10.4 [−1175.4; 1154.7]	0.985	−0.02	2183.3 [−50.2; 4416.7]	0.054	2.31	0.576	0.27
HF (nu)	0.46 [−7.45; 8.36]	>0.999	−0.03	18.66 [9.39; 27.93]	**0.001**	−0.97	**0.025**	−1.01
LF/HF ratio	0.08 [−0.55; 0.71]	>0.999	−0.09	−1.02 [−1.76; −0.28]	**0.020**	0.92	**0.007**	1.17
TP (ms^2^)	−202.0 [−1831.0; 1427.0]	0.788	−0.08	1872.5 [−1843.4; 5588.4]	0.272	0.42	**0.025**	−1.01
SD1 (ms)	−2.31 [−15.52; 10.90]	>0.999	0.11	13.46 [−2.03; 28.95]	0.169	−0.37	0.178	−0.64
SD2 (ms)	−5.67 [−23.89; 12.55]	>0.999	0.18	12.56 [−8.80; 33.93]	0.463	−0.28	0.221	−0.58
SD2/SD1 ratio	−0.08 [−0.33; 0.18]	>0.999	0.33	−0.13 [−0.43; 0.17]	0.764	0.23	0.839	0.10

*D, raw difference pre-post; [95% CI], 95% confidence interval; d, Cohen’s d standardized effect size; p-values for D were calculated using one sample t-tests; between-group differences in % change were calculated using independent samples t-tests. ms, milliseconds; nu, normalized units; SDNN, standard deviation of R-R intervals; RMSSD, root mean square of successive differences between normal heartbeats; PNN50, percentage of successive R-R intervals that differ by more than 50 ms; LF, low frequency band; HF, high frequency band; SD1, dispersion of points perpendicular to the short-term identity line; SD2, dispersion of points along the identity line. Bold values indicate significant differences (*p* < 0.05).*

## Discussion

The results of the present study confirmed our hypothesis that an 8-week resistance exercise program is able to modulate HRV in children with CF. To the best of our knowledge, this is the first study to investigate the influence of an exercise program on HVR in children and adolescents with CF.

HRV has been commonly applied to high-performance athletes in order to control working load doses during training periods (Manzi et al., 2009). In addition, other areas, such as nutrition, have also studied the influence of ergogenic supplements and its association with HRV (Zimmermann-Viehoff et al., 2016). Psychological factors as stress or anxiety have been related to sympatho-vagal balance (Goessl et al., 2017). On the other hand, HRV may also play an important role in clinical settings, as a complementary tool to extract information on the diagnosis and evolution of several diseases, including Diabetes (Gutin et al., 1997), Asthma (McNarry et al., 2019), and CF (McNarry and Mackintosh, 2016). Although HRV is well-reported in several studies with adults (Nunan et al., 2010), there is considerably less evidence available in children. The evaluation of the sympatho-vagal balance in children with CF, measured through HRV, was associated with a good to excellent reproducibility and a useful prognostic tool (McNarry and Mackintosh, 2016). On the other hand, we believe it is important to note that the use of LF/HF to evaluate sympatho-vagal balance is still a matter of debate, as the utility of LF HRV as an index of sympathetic cardiac tone has been questioned (Billman, 2013; Reyes del Paso et al., 2013). Considering that autonomic function is altered in people with CF (Davis and Kaliner, 1983) and that exercise may be a regulator of its function (Caruso et al., 2015), the present study focused on the effects of an exercise program and have used HRV to provide an indirect assessment of cardiac autonomic activity, rather than a direct measure of cardiac parasympathetic or sympathetic nerve activity. In spite of that, normal values for HRV have been described for the healthy children population. CF children exhibit a sympathetic dominance at rest compared to healthy children, which persisted after a 6-min walk test (Florêncio et al., 2013). Resting values described by Florêncio et al. (2013) were comparable to the ones found in the present study. Although differences regarding the recording tool and protocol exists between studies, our results have shown that the majority (57.9%) of individuals with CF presented at least one altered HRV variable when data was normalized using reference values for healthy children (Gasior et al., 2018).

Evidence on the influence of exercise upon the ANS in healthy children is limited. In 2009, a study using 7 weeks of high aerobic intensity training (HIIT) was not able to demonstrate influence on the heart rate autonomic regulation (Gamelin et al., 2009). However, 13 weeks of aerobic exercise had a positive effect on HRV global cardiac parameters, although this improvement was seen during nocturnal long-term recordings (Mandigout et al., 2002). Studies have also evaluated the effects of exercise on HRV in children with several diseases (Prado et al., 2010; McNarry et al., 2019). Our results have shown that a resistance exercise-training program was able to modulate the sympatho-vagal balance in children with CF. To the best of our knowledge, no studies have investigated the effects of an exercise program focused on resistance training and its influence on the ANS in children and adolescents with CF. However, evidence in obese children shows that a 12-week resistance training program also improved HRV (Farinatti et al., 2016). In addition, a recent study has shown the effectiveness of a 5-week multimodal program (aerobic and resistance training combined with diet) for obese adolescents on HRV parameters (Huang et al., 2019). On the other hand, the effects of aerobic exercise-training on HRV have been widely explored in obese children (Farah et al., 2014) and most of the studies concluded that training was able to change the cardiac autonomic function by reducing the sympathetic activity (Gutin et al., 1997). Taken together, we believe it is important to highlight that comparisons must be interpreted with caution, as baseline mechanisms leading to altered HRV may differ between obese and CF individuals, and several factors may have influenced results, including the length of exercise programs, the influence of diet, among others. As for chronic respiratory diseases, evidence has shown that a 6-month HIIT in adolescents with asthma produced no effects in both time-domain and non-linear indices (Prado et al., 2010; McNarry et al., 2019). No studies on the effects of exercise training on HRV in individuals with CF were found for comparisons.

Physical activity and exercise programs have been largely used in children with CF as a tool to improve general physical health, morbi-mortality and exercise tolerance (Radtke et al., 2017; Vendrusculo et al., 2019). The majority of studies evaluating the effects of exercise training in children were performed using aerobic exercise programs (Gutin and Owens, 1999; Huang et al., 2019), in contrast to the present study, in which the exercise program was based on resistance training. Evidence evaluating the effects of resistance exercise for individuals with CF have shown positive results in both aerobic and anaerobic performance, health-related quality of life and lung function (Kriemler et al., 2013; Santana-Sosa et al., 2014). Our results have shown that an 8-week resistance-training program was able to improve the sympatho-vagal balance. Considering the well-known compromise of peripheral muscles in individuals with CF, including decreased muscle strength (Gruet et al., 2017), reduction of contractile excitability (Clay, 2013), cellular alterations (Antigny et al., 2009), and loss of muscle mass, it is possible that the resistance-training would improve muscle performance leading to the several benefits already described, including the modulation of the ANS.

The present study also has limitations, including the reduced sample size, which may have prevented time-domain variables from reaching statistical significance, although a moderate effect size was seen. The inclusion of a sample of children with CF and high aerobic fitness (mean VO_2_peak of 42.7 mL.Kg^–1^.min^–1^) may have also decreased the effects of an exercise-based program. In spite of that, significant improvements were seen on the modulation of the ANS. The use of long-term HRV protocols could improve the precision of data collection, although its application decreases clinical practicability in routine CF management and short-term protocols have been validated. Respiratory rate is known to influence HRV, specially the HF band, and although breathing pattern was not controlled, patients remained in a quiet environment, without speaking or performing any important body movements, and no differences between baseline respiratory rate were seen between groups.

## Conclusion

Our findings support the effectiveness of a short-term exercise resistance training program to modulate HRV in children and adolescents with CF presenting mild to moderate lung function impairment and a good physical state.

## Data Availability Statement

The raw data supporting the conclusions of this article will be made available by the authors, without undue reservation.

## Ethics Statement

The studies involving human participants were reviewed and approved by Hospital Universitario Niño Jesús de Madrid and Universidad Europea de Madrid. Written informed consent to participate in this study was provided by the participants’ legal guardian/next of kin.

## Author Contributions

AE-G, MD, and FC-V: literature search. AE-G, FC-V, ÁF-L, TI, and MF-d-V: data collection. MP-R, MD, VS-S, and JV: study design. ID-V, EL-Z, AE-G, and MD: analysis of data. AE-G and MD: manuscript preparation. MP-R, EL-Z, and MD: final critical review. All authors read and approved the final version of the manuscript.

## Conflict of Interest

The authors declare that the research was conducted in the absence of any commercial or financial relationships that could be construed as a potential conflict of interest.

## Supplementary Material

Supplementary Figure 1 Resistance training exercises used in the intervention-training program. **(A)** Leg press; **(B)** bilateral seated row; **(C)** leg extension; **(D)** seated bench press; **(E)** leg flexion.
