# Supplementary figures and images for: Effects of a Short-Term Resistance-Training Program on Heart Rate Variability in Children With Cystic Fibrosis—A Randomized Controlled Trial
Source: Front Physiol. 2021 Mar 30;12:652029. doi: 10.3389/fphys.2021.652029 (PMC8042150; doi:10.3389/fphys.2021.652029)

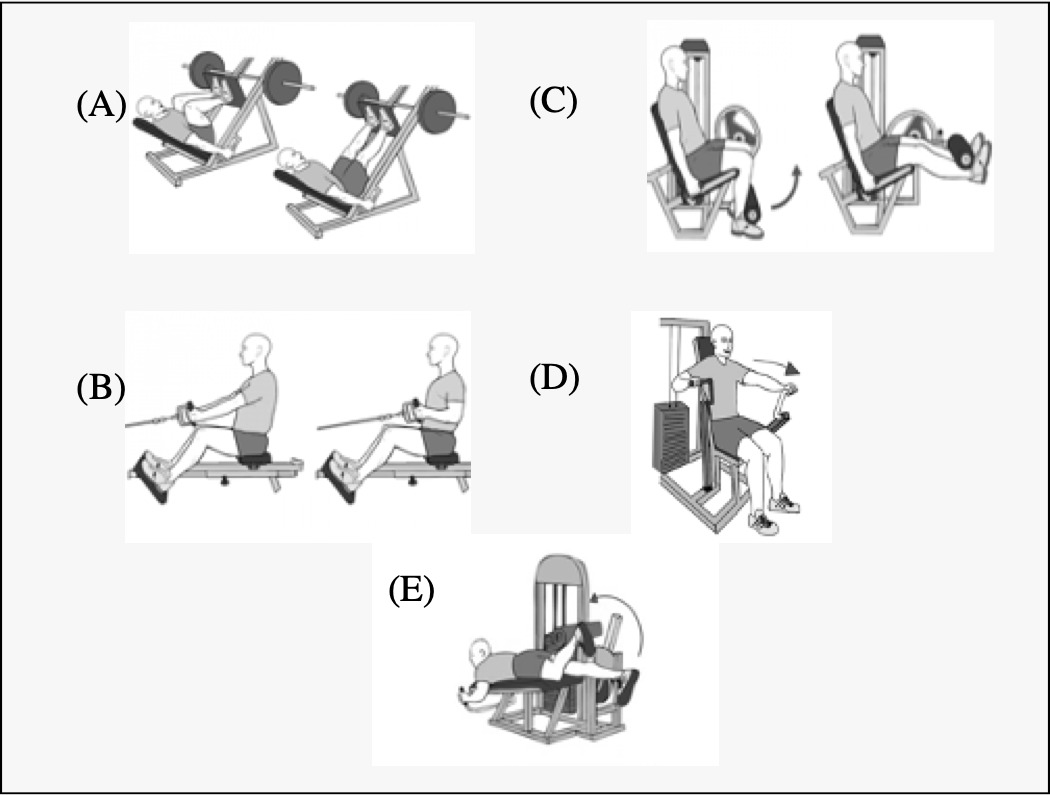

Supplement: Supplementary Figure 1 — Resistance training exercises used in the intervention-training program. (A) Leg press; (B) bilateral seated row; (C) leg extension; (D) seated bench press; (E) leg flexion. [file Image_1.tif]
